# Expert assessment of ChatGPT’s ability to generate illness scripts: an evaluative study

**DOI:** 10.1186/s12909-024-05534-8

**Published:** 2024-05-15

**Authors:** Yasutaka Yanagita, Daiki Yokokawa, Fumitoshi Fukuzawa, Shun Uchida, Takanori Uehara, Masatomi Ikusaka

**Affiliations:** 1 Department of General Medicine, Chiba University Hospital, 1-8-1, Inohana, Chuo-Ku, Chiba, Chiba Pref Japan; 2 Uchida Internal Medicine Clinic, Saitama, Japan

**Keywords:** ChatGPT-4, Generative pretrained transformer (GPT), Illness script, Medical education, AI

## Abstract

**Background:**

An illness script is a specific script format geared to represent patient-oriented clinical knowledge organized around enabling conditions, faults (i.e., pathophysiological process), and consequences. Generative artificial intelligence (AI) stands out as an educational aid in continuing medical education. The effortless creation of a typical illness script by generative AI could help the comprehension of key features of diseases and increase diagnostic accuracy. No systematic summary of specific examples of illness scripts has been reported since illness scripts are unique to each physician.

**Objective:**

This study investigated whether generative AI can generate illness scripts.

**Methods:**

We utilized ChatGPT-4, a generative AI, to create illness scripts for 184 diseases based on the diseases and conditions integral to the National Model Core Curriculum in Japan for undergraduate medical education (2022 revised edition) and primary care specialist training in Japan. Three physicians applied a three-tier grading scale: “A” denotes that the content of each disease’s illness script proves sufficient for training medical students, “B” denotes that it is partially lacking but acceptable, and “C” denotes that it is deficient in multiple respects.

**Results:**

By leveraging ChatGPT-4, we successfully generated each component of the illness script for 184 diseases without any omission. The illness scripts received “A,” “B,” and “C” ratings of 56.0% (103/184), 28.3% (52/184), and 15.8% (29/184), respectively.

**Conclusion:**

Useful illness scripts were seamlessly and instantaneously created using ChatGPT-4 by employing prompts appropriate for medical students. The technology-driven illness script is a valuable tool for introducing medical students to key features of diseases.

**Supplementary Information:**

The online version contains supplementary material available at 10.1186/s12909-024-05534-8.

## Introduction

An illness script is defined as a specific script format geared to represent patient-oriented clinical knowledge organized around enabling conditions, faults (i.e., pathophysiological process), and consequences [1]. This script encompasses key elements of diseases. such as pathophysiology, epidemiology, time course, symptoms and signs, diagnosis, and treatment [2]. An illness script is the cognitive structure of a clinician's knowledge of a disease, which is formed based on personal experience, formal learning, and clinical practice. This allows physicians to efficiently organize and pull complex information from memory to aid in diagnosis [1, 3].

Reports suggest that leveraging illness scripts can improve the instruction of clinical reasoning and serve as an effective method for refining the learner’s clinical reasoning skills [4–6]. Therefore, illness scripts increase diagnostic accuracy and are useful for continuing medical education [7].

Conversely, the clinical application of illness scripts is not straightforward. Clinicians iteratively enhance their illness scripts through their clinical practice and by encountering various cases, including those considered atypical. Illness scripts are not static, that is, they refine and develop as clinicians enhance their skills.

Therefore, no standardized illness scripts exist for any disease, and creating them for educational purposes is time-consuming. Hence, our focus on large language models (LLMs), is due to the notable progress achieved in natural language processing using generative pretrained transformers (GPT) [8]. Although generative AI, as typified by ChatGPT-4, was not explicitly designed for medical applications, previous research has showcased ChatGPT-4’s capability to successfully pass medical licensing examinations in the United States and Japan [9, 10]. It has contributed to generating differential diagnosis lists from patient histories [11], clinical vignettes [12], and intervened in various aspects of medicine. Furthermore, the potential of AI models in specialized medical education and practice is acknowledged [13]. ChatGPT was utilized to generate initial drafts of United States Medical Licensing Examination-style, multiple-choice items [14].

With the rapid progress of AI technology, if a generative AI tool, such as ChatGPT-4, could be used to generate illness scripts that are beneficial in understanding the key features of diseases, this could be applied to medical education.

No research has delved into the automated generation of illness scripts tailored to individual diseases. Furthermore, the output’s accuracy becomes critical when integrating such technologies into the medical domain due to the implications for disease diagnosis and treatment. This study attempts to investigate that ChatGPT can create an illness script that contains sufficient information for medical students to learn about diseases. Since ChatGPT-4 is known to generate incorrect information, three board-certified physicians assessed whether ChatGPT-4 can adeptly generate an illness script containing adequate information.

## Methods

### Study design

Focusing on the illnesses and conditions integral to the Japanese National Model Core Curriculum for undergraduate medical education (2022 revised edition) [15] and primary care training program in Japan [16], the illness scripts for 184 diseases were systematically generated using ChatGPT-4. Subsequently, three board-certified general physicians (YY, SU, and FF) assessed if the generated output reached the level required for graduating medical students. Finally, each illness script was graded on a three-point scale, that is, “A” denotes that the content proved sufficient for medical students, “B” denoted that it exhibited partial inadequacy, and “C” denotes that it was deemed inadequate in multiple aspects.

### Large language model environment

The illness scripts were generated on July 25, 2023, using the July 20 version of GPT-4 (OpenAI, San Francisco, California, USA). GPT is a large language model (LLM) developed by OpenAI for natural language processing. Its dynamic response generation is based on probabilities the neural network derives from learned syntactic and semantic relationships in the text [17].

### Selecting diseases for illness scripts

Commonly and frequently encountered diseases were selected due to their importance for medical students. Considering that the diseases managed in primary care overlap with those that medical students should learn about, the diseases studied in primary care training in Japan [16] were used as a reference. Among the 205 disease and symptom items representing the 16 areas targeted for appropriate management in primary care [16], 184 were identified as sufficiently relevant for the creation of the illness script. These diseases are included in the National Model Core Curriculum in Japan for undergraduate medical education (2022 revised edition) [15].

Physicians YY, SU, and FF established the exclusion criteria through collaborative discussions and excluded 21 items with minimal diagnostic contribution or mere symptomatology. Seventeen items (e.g., those associated with palliative care or non-critical symptoms, such as lower back pain) were omitted because they lacked the specificity for script creation. Furthermore, four items related to community-acquired pneumonia, herpes encephalitis, herpes infections, and adrenal insufficiency were excluded because they were pertinent to the input examples in the prompt. The English names for the 184 selected items were entered into the prompt based on the International Classification of Diseases, 11th Revision (ICD-11) [18] registered disease names (Supplementary Material).

### Content to be entered into ChatGPT-4, program code

**Fig. 1:**
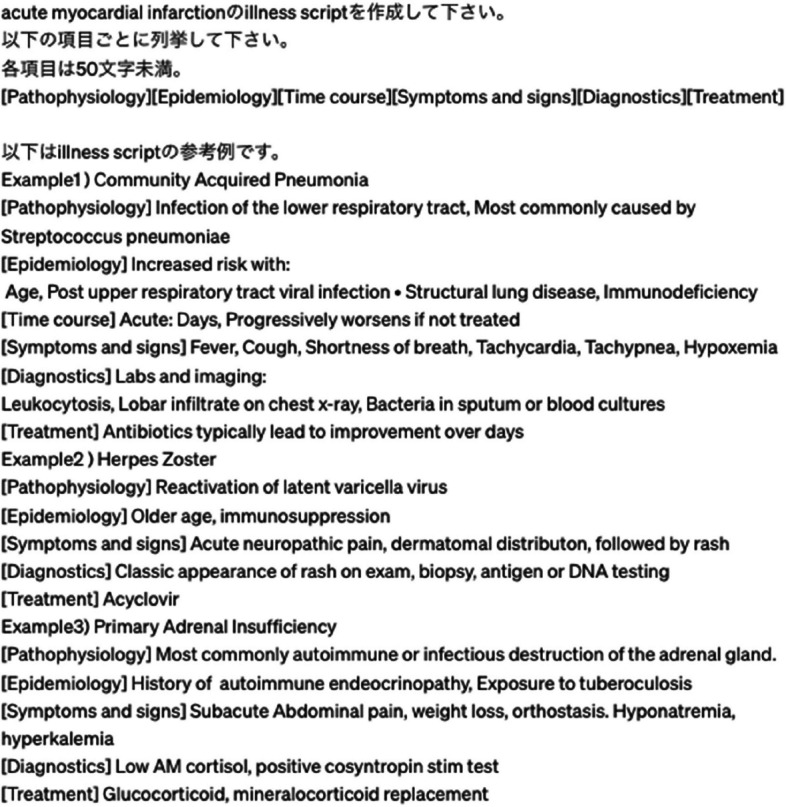
Screenshot of the prompt input

The prompts for ChatGPT-4 were carefully engineered to ensure their interpretability by generative AI while succinctly defining the desired outputs [19]. The output items referencing the proposed elements of illness scripts [2] were determined after discussions facilitated by one board-certified physician (YY) and fellow of internal medicine (DY). The input-specified key elements of the illness scripts included pathophysiology, epidemiology, time course, signs and symptoms, diagnosis, and treatment. The character limit per item was set at less than 50 characters, based on findings from prior illness scripts [2] and the general requirement that an average of 20–30 words per English sentence could be generated. Three output examples (community-acquired pneumonia, herpes zoster, and primary adrenal insufficiency) were added after key elements. The structured prompt for ChatGPT-4 was: [Create an illness script for < disease name > . List the following items in less than 50 characters each: [pathophysiology][epidemiology][time course][Symptoms and Signs][Diagnostics][and treatment]. The following is a reference example of an illness script. Example1), Example2), Example3)] (Fig. 1). This prompt was entered into ChatGPT once, and the output information was evaluated. No additional prompts were entered to indicate modifications.

### Evaluation

A broader evaluation was conducted by physicians YY, SU, and FF to assess the generated illness script’s utility for medical students.

Following a discussion among the three evaluators, the usefulness of the illness scripts in this study was defined as the level at which each item contained the minimum amount of required information and would not cause inconvenience to a medical student learning to use the illness scripts for the first time. Initially, screening was conducted by physician YY to ensure that the output included the essential elements of the illness script: pathophysiology, epidemiology, time course, symptoms and signs, diagnosis, and treatment. Subsequently, the three evaluators rated the illness scripts with all output items on a five-point scale. The evaluation was structured on a five-point scale, where 1 denotes “not at all useful, needs overall revision,” and 5 denotes “very useful, no additional modifications needed.” To achieve a structured assessment, each item was evaluated considering the age and mode of onset, typical symptoms, essential diagnostic examinations, standard treatment, and adequacy of the course of treatment. Failure to meet these items resulted in a point deduction. The rating of each evaluator was summed, and each illness script was scored on a 15-point scale. Composite scores were categorized into three levels: 15, 14, and 13 or less, corresponding to “A,” “B,” and “C,” respectively. Moreover, any identified deficiencies in the illness scripts were discussed during the evaluation. Consequently, an “A” rating signifies a script that proved sufficiently informative for medical students and required no further modification, “B” is a script that was partially sufficient or required minor revision but was acceptable. “C” represents a script that was inadequate in several respects and necessitated multiple revisions. Then, we discussed the reasons for discrepancies in the evaluations and identified the main aspects that were lacking in the creation of the illness scripts by ChatGPT, along with considerations for their educational application.

### Ethical considerations

This study did not involve human or animal participants, thereby obviating the need for ethical approval.

## Results

**Fig. 2:**
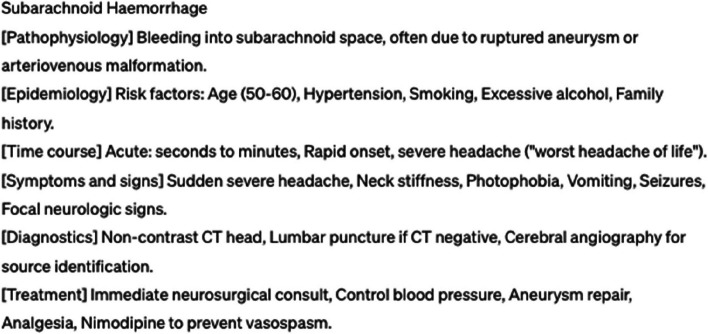
Example of an A-rated illness script

**Fig. 3:**
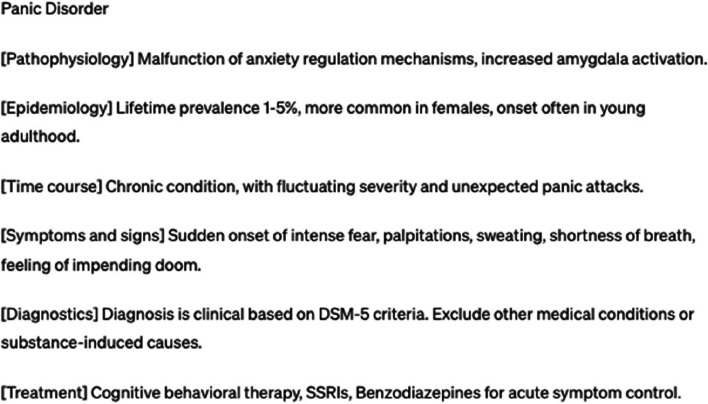
Example of a C-rated illness script

The illness scripts for all 184 target diseases were successfully generated using ChatGPT-4. Each script incorporated the following specified elements: pathophysiology, epidemiology, time course, signs and symptoms, diagnosis, and treatment, strictly following the prompt instructions. The distribution of the ratings among the evaluated scripts revealed that 56.0% (103/184) were rated “A,” 28.3% (52/184) were “B,” and 15.8% (29/184) were “C”. Examples of the illness scripts with “A” and “C” ratings are presented in Figs. 2 and 3, respectively. Table 1 lists the breakdown of evaluations across different categories. Cardiovascular and psychiatric systems had the highest number of “C” ratings.

**Table 1:** Distribution of ratings by 16 areas

Classification	Rate A (n (%))	Rate B (n (%))	Rate C (n (%))	Total
Hematological system	1 (20.0)	3 (60.0)	1 (20.0)	5
Neurological system	2 (20.0)	5 (50.0)	3 (30.0)	10
Dermatological system	8 (61.5)	5 (38.5)	0 (0.0)	13
Musculoskeletal System	8 (80.0)	1 (10.0)	1 (10.0)	10
Cardiovascular System	6 (42.9)	2 (14.3)	6 (42.9)	14
Respiratory System	8 (72.7)	3 (27.3)	0 (0.0)	11
Gastrointestinal System	17 (68.0)	7 (28.0)	1 (4.0)	25
Renal and Urinary system	3 (33.3)	5 (55.6)	1 (11.1)	9
Pregnancy and Reproductive System	10 (66.7)	3 (20.0)	2 (13.3)	15
Endocrine, Nutritional, and Metabolic System	2 (25.0)	4 (50.0)	2 (25.0)	8
Ophthalmological and Visual System	6 (75.0)	1 (12.5)	1 (12.5)	8
Otorhinolaryngological and Oral Cavity	9 (90.0)	0 (0.0)	1 (10.0)	10
Psychiatric System	4 (36.4)	2 (18.2)	5 (45.5)	11
Infectious	3 (30.0)	4 (40.0)	3 (30.0)	10
immunologic and Allergic	1 (50.0)	1 (50.0)	0 (0.0)	2
Physical and Chemical Factors	3 (50.0)	3 (50.0)	0 (0.0)	6
Pediatric	10 (66.7)	4 (26.7)	1 (6.7)	15
Geriatrics	2 (100.0)	0 (0.0)	0 (0.0)	2
Total	103 (56.0)	52 (28.3)	29 (15.8)	184

Deficiencies in the output of ChatGPT-4’s illness scripts were identified during a comprehensive discussion among physicians YY, SU, and FF, focusing on the deduced points. The deficiencies identified within each component of the illness script are outlined as follows:

Pathophysiology:

1. Droplet transmission for varicella was incorrectly indicated as a route of infection.

Epidemiology:

1. The phrase “Risk: Age” was unclear regarding the specific age group to which it referred.
2. Genetic diseases, such as the von Willebrand disease, lacked the associated family history.
3. Phrases, such as “more common in certain ethnic groups”, were deemed too vague.

Time course:

1. The duration of a single attack for cluster headaches was not mentioned besides the symptomatic period.

Diagnostics:

1. Outputs were criticized for being too generic, such as “refer to guidelines” or “exclude similar conditions.”
2. In mitral valve insufficiency and aortic valve stenosis, “Heart murmur on auscultation” was described but the type of murmur was not specified.

Treatment:

1. A paper bag (no longer recommended, especially for adults) was generated for hyperventilation syndrome.
2. Inappropriate antibiotic treatment was generated for non-purulent mastitis.
3. The use of the abbreviation VNS was noted for treating epileptic encephalopathy.
4. The treatment for tension headaches is listed as NSAIDs or triptans, but only “use prophylaxis if the frequency is high.” Limited and contradictory data exist concerning the effectiveness of triptans in treating tension-type headaches.
5. In abdominal aortic aneurysms, the description is “monitor small aneurysms, surgical repair (open or endovascular) for large or rapidly growing aneurysms.” The definitions of small and large are unclear.

## Discussion

We utilized ChatGPT-4 to construct easily comprehensible illness scripts for 184 diseases based on the topics covered in the Japanese National Model Core Curriculum for undergraduate medical education (2022 revised edition) [15] and primary care residency programs in Japan [16]. The three physicians assigned an “A” rating to 56% of the developed illness scripts, signifying their adequacy. More than half of the generated illness scripts required no changes. Furthermore, 28.3% were rated “B,” indicating partial sufficiency with potential usability after minor revisions and additions. The “A” and “B” ratings (i.e., approximately 84% of the illness scripts) demonstrated relatively high accuracy.

The illness scripts rated as “B” exhibited specific characteristics, such as omitting family history as a crucial risk factor for genetic diseases. In the case of tension headache, the preventive treatment was only indicated if the frequency was high, suggesting the need for more specificity in the output. However, given that one reviewer found the content partially insufficient while the others deemed it sufficient, the overall content was arguably adequate for medical students. A potential solution to address this variability is by adjusting the content of prompts (e.g., character limit restrictions) [19].

The illness scripts receiving a "C" rating lacked critical information for diagnosis, such as missing essential symptoms or tests. The rater observed that the valvular disease illness script should describe the presence of a heart murmur and the type of murmur. This assessment indicates a potential influence from the learning material on which the generative AI was trained [20]. The information on the web on valvular disease is expected to be described only in the presence or absence of a heart murmur, leading to inadequate AI output. These errors can occur in a certain percentage of outputs when generating large volumes of content.

Notably, the prevalence of “A” and “B” ratings was observed on the 16 areas regarding the accuracy of illness scripts across different diseases. However, the cardiovascular and psychiatric scripts exhibited a higher proportion of “C” ratings. A more explicit description was required to treat abdominal aortic aneurysms because of variations in the treatment approaches, which is based on the aneurysm size. In the psychiatric system, outputs, such as “diagnosis based primarily on clinical interview and symptom criteria (DSM-5)”, were considered considerably general and lacking specificity. Constraints on the item’s character count may have contributed to the challenge of providing detailed information, particularly given the multifaceted nature of cardiovascular assessments and the wide variety of psychiatric symptoms. Analyzing the illness scripts’ outputs, we could observe that the compilation of symptoms and tests did not consider the frequency of symptoms or the sensitivity of the tests.

This study employed a straightforward input approach to ChatGPT-4, specifying three examples of illness scripts in the prompt to control the output standard. To ensure that each item was within 20–30 words, a pilot study was conducted for verification, and the prompt was set to input 50 Japanese characters. As a result, there was one item that had a maximum length of 22 words, while the other items were within 20 words. However, several illness scripts lacked essential details since the character limits were set for each item to minimize redundant information. Adjusting the character limits for prompts or prioritizing symptoms based on frequency could improve the output for specific diseases or conditions. Furthermore, modifying the character count may allow more accurate illness scripts to be created, especially for complex systems, such as the cardiovascular system, which have many “C” ratings. Moreover, several methods are employed for refining the scripts, including changing the number of characters for each item and specifying the data to be referenced in the interactive exchange between the script and ChatGPT [21].

Previous studies reported that various medical information generated using AI has been reported and is increasingly applied to the medical field [22, 23]. Considering this, the illness scripts generated in this study can be used in the field of medical education in the future. A previous study reported that clinical vignettes created by ChatGPT are comparable to those created by human authors [24]. Although concerns persist regarding copyright issues and the medical accuracy of content generated by generative AI systems [25], careful consideration and appropriate use can significantly expand their utility. Medical educators can curate outputs, enabling generative AI to be utilized in delivering educational information to students [26]. This tool would reduce the burden on educators, who could focus on other educational content. In addition, students may be able to create content based on their own level of understanding by teaching them how to generate illness scripts. However, greatly relying on such convenience may lead to various issues. The biggest disadvantage of using such systems is that students may believe in all the output content without critically analyzing its accuracy [27].

Extending the illness script concept [28] to other potential applications across the healthcare field is presently being investigated. We expect that more accurate illness script generation will be achieved in the future, and the prompt of ChatGPT may be customized by the educators [29]. AI could play a pivotal role in providing valuable insights and information by generating these extended illness scripts.

### Limitation

This study has several limitations. First, the evaluation was conducted based on the GPT-4 version available from July 25, 2023. Given that further updates are anticipated, continuous evaluation is necessary.

Second, the absence of clear standards for evaluating illness scripts is noteworthy. This study relied on the subjective assessments of three physicians, and results might vary if evaluated by physicians from other specialties.

Third, the utility was evaluated based on the potential benefit of illness scripts to medical students. We did not verify the usefulness for educators or specialists in various fields, indicating an avenue for future research.

## Conclusion

Generative AI enables the swift and seamless production of illness scripts. While the results must be carefully reviewed, the potential applications of this technology in medical education are evident. AI-generated illness scripts are useful for medical students and for developing and refining their own scripts.

### Supplementary Information

**Supplementary Material 1. **

## Data Availability

Data on the results of this study are available from the corresponding author (YY) upon reasonable request.

## Abbreviations

AI: Artificial intelligence
LLM: Large language models
GPT: Generative pretrained transformer
ICD-11: International classification of diseases 11th revision
DSM-5: Diagnostic and statistical manual of mental disorders-5

## References

[1] Custers EJ (2015). Thirty years of illness scripts: Theoretical origins and practical applications. Med Teach.

[2] Jones B, Brzezinski WA, Estrada CA, Rodriguez M, Kraemer RR (2014). A 22-year-old woman with abdominal pain. J Gen Intern Med.

[3] Bowen JL (2006). Educational strategies to promote clinical diagnostic reasoning. N Engl J Med.

[4] Maciuba JM, Mallory R, Surry L (2023). Teaching students how to think: A longitudinal qualitative study of Preclerkship clinical reasoning instruction. Mil Med.

[5] Lee A, Joynt GM, Lee AK (2010). Using illness scripts to teach clinical reasoning skills to medical students. Fam Med.

[6] Moghadami M, Amini M, Moghadami M, Dalal B, Charlin B (2021). Teaching clinical reasoning to undergraduate medical students by illness script method: A randomized controlled trial. BMC Med Educ.

[7] Oliveira JCV, Peixoto AB, Marinho GEM, Peixoto JM. Teaching of Clinical Reasoning Guided by Illness Script Theory. Ensino do Raciocínio Clínico Orientado pela Teoria dos Scripts de Doenças. Arq Bras Cardiol. 2022;119(5 suppl 1):14–21. 10.36660/abc.20220419. 10.36660/abc.20220419 PMC9750199 36449954

[8] Open AI. GPT-4 Technical Report. arXiv. 2023. 10.48550/arXiv.2303.08774.

[9] Gilson A, Safranek CW, Huang T (2023). How does ChatGPT perform on the United States medical licensing examination? The implications of large language models for medical education and knowledge assessment. JMIR Med Educ.

[10] Yanagita Y, Yokokawa D, Uchida S, Tawara J, Ikusaka M (2023). Accuracy of ChatGPT on medical questions in the national medical licensing examination in Japan: Evaluation study. JMIR Form Res.

[11] Hirosawa T, Harada Y, Yokose M, Sakamoto T, Kawamura R, Shimizu T (2023). Diagnostic accuracy of differential-diagnosis lists generated by generative pretrained Transformer 3 Chatbot for Clinical Vignettes with Common Chief Complaints: A Pilot Study. Int J Environ Res Public Health.

[12] Bakkum MJ, Hartjes MG, Piët JD (2024). Using artificial intelligence to create diverse and inclusive medical case vignettes for education. Br J Clin Pharmacol.

[13] Giannos P (2023). Evaluating the limits of AI in medical specialisation: ChatGPT's performance on the UK Neurology Specialty Certificate Examination. BMJ Neurol Open.

[14] Zuckerman M, Flood R, Tan RJB (2023). ChatGPT for assessment writing. Med Teach.

[15] Medical Education Model Core Curriculum Coordination Committee. Medical education model core curriculum expert research committee. Model core curriculum for medical education. AY 2022 Revision. Available at: https://www.mext.go.jp/b_menu/shingi/chousa/koutou/116/toushin/mext_01280.html. Accessed December 21, 2023.

[16] Japan Primary Care Association. Specialty Training Programs [homepage on the Internet]. https://www.primary-care.or.jp/nintei_tr/kouki_touroku.php. Accessed June 22, 2023. Accessed June 22, 2023.

[17] Chat GPT. Optimizing language models for dialogue [homepage on the Internet]. https://openai.com/blog/chatgpt/. Accessed on June 22, 2023.

[18] International Classification of Diseases. 11th revision [homepage on the Internet]. https://icd.who.int/en. Accessed June 22, 2023. Accessed June 22, 2023.

[19] White J, Fu Quchen, Hays S, et al. A Prompt Pattern Catalog to Enhance Prompt Engineering with ChatGPT. arXiv:2302.11382.

[20] Vaishya R, Misra A, Vaish A (2023). ChatGPT: Is this version good for healthcare and research?. Diabetes Metab Syndr.

[21] Long O, Jeff W, Xu J, et al. Training language models to follow instructions with human feedback. arXiv. 2022. 10.48550/arXiv.2203.02155.

[22] Wong RS, Ming LC, Raja Ali RA (2023). The Intersection of ChatGPT, Clinical Medicine, and Medical Education. JMIR Med Educ.

[23] Lee H. The rise of ChatGPT: Exploring its potential in medical education. Anat Sci Educ. 2023 March 14. 10.1002/ase.2270. Epub ahead of print. PMID: 36916887. 10.1002/ase.2270 36916887

[24] Coşkun Ö, Kıyak YS, Budakoğlu Iİ. ChatGPT to generate clinical vignettes for teaching and multiple-choice questions for assessment: A randomized controlled experiment. Med Teach. Published online March 13, 2024. 10.1080/0142159X.2024.2327477. 10.1080/0142159X.2024.2327477 38478902

[25] Dave T, Athaluri SA, Singh S (2023). ChatGPT in medicine: An overview of its applications, advantages, limitations, future prospects, and ethical considerations. Front Artif Intell.

[26] Meşe İ, Altıntaş Taşlıçay C, Kuzan BN, Kuzan TY, Sivrioğlu AK. Educating the next generation of radiologists: a comparative report of ChatGPT and e-learning resources. Diagn Interv Radiol. Published online December 25, 2023. 10.4274/dir.2023.232496. 10.4274/dir.2023.232496 PMC11095068 38145370

[27] Mu Y, He D (2024). The Potential Applications and Challenges of ChatGPT in the Medical Field. Int J Gen Med.

[28] Vreugdenhil J, Döpp D, Custers EJFM, Reinders ME, Dobber J, Kusukar RA (2022). Illness scripts in nursing: Directed content analysis. J Adv Nurs.

[29] Masters K, Benjamin J, Agrawal A, MacNeill H, Pillow MT, Mehta N. Twelve tips on creating and using custom GPTs to enhance health professions education. Med Teach. Published online January 29, 2024. 10.1080/0142159X.2024.2305365. 10.1080/0142159X.2024.2305365 38285894

